# Transformation of the cyclohexane ring to the cyclopentane fragment of biologically active compounds

**DOI:** 10.3762/bjoc.21.185

**Published:** 2025-11-06

**Authors:** Natalya Akhmetdinova, Ilgiz Biktagirov, Liliya Kh Faizullina

**Affiliations:** 1 Ufa Institute of Chemistry, UFRC RAS, 450054, Ufa, 71, Oktyabrya Ave. Russian Federation; 2 «Bazis-Terra» LLC, 20a, Golubyatnikova St., Kazan, 420094, Russian Federation

**Keywords:** biological activity, cyclopentane/enone, rearrangements, ring/cycle contractions, total synthesis

## Abstract

The review is devoted to strategies for contraction of six-membered cycles in the synthesis of functionalized cyclopentane/enones, which are biologically active compounds. The main synthetic methods of ring contraction (ozonolysis–aldol condensation, ozonolysis–Dieckmann reaction, Baeyer–Villiger cleavage–Dieckmann reaction) and rearrangements (benzil, semipinacol, with the participation of thallium- and iodine-based oxidants, photochemical, Wolff, Meinwald, Wagner–Meerwein and Favorskii) are presented. The review summarizes literature data covering the last 12 years, with some exceptions of earlier works due to the importance of the published information.

## Introduction

The functionalized cyclopentane/enone fragment is part of the structure of a large number of natural and synthetic biologically active compounds, including prostaglandins, terpenoids, alkaloids, steroids and carbanucleosides. The spectrum of biological activities of the listed classes is wide and unique in efficacy. Natural prostaglandins are polyoxygenated derivatives of a hypothetical twenty-atom prostanoic acid, the C8–C12 atoms of which are contained in a cyclopentane ring with two attached side chains – of seven (α-chain) and eight (β- or ω-chain) carbon atoms. Their main physiological role is to maintain the homeostatic harmony of the organism [1–2]. Among the terpenoids containing cyclopentane in their structure, we should mention jatrophane or latirane diterpenoids, in which the five-membered cycle is annelated with a functionally saturated dodecyl fragment [3–4], as well as iridoids – bicyclic compounds with a central cyclopentane unit [5]. Estrone (folliculin, estra-1,3,5(10)-trien-3-ol-17-one), a steroid containing a cyclopentane annelated with octahydrophenanthrene, is a natural follicular hormone essential for normal development of the female body [6]. An example of an alkaloid whose structure includes cyclopentane is lappaconitine, which is noteworthy because its hydrobromide is the active ingredient in the highly effective antiarrhythmic drug allapinine [7].

Syntheses of cyclopentanoids usually are based on cyclopentaannelation reactions:

- transformations accompanied by cyclization [8];
- transformation of compounds containing a cyclopentane ring [9];
- contraction of large cycles to cyclopentane derivatives.

Ring contraction reactions are among the most useful strategic transformations for the construction of carbocyclic and heterocyclic cyclopentanes. Six-membered carbocycles are convenient starting compounds for the synthesis of cyclopentanoids due to their widespread occurrence in nature and their synthetic availability. Undoubtedly, the development of practical approaches to the synthesis of known compounds and the synthesis of new biologically active functionalized compounds containing a cyclopentane/enone fragment through transformation of a cyclohexane/ene ring is an urgent task for synthetic chemists.

This review summarizes information on cyclohexane/ene ring contraction. The structure of the review includes examples of simple transformations (ozonolysis–aldol condensation, ozonolysis–Dieckmann reaction, and Baeyer–Villiger cleavage–Dieckmann reaction) and rearrangements (photochemical, benzil, semi-pinacol, Wolff, Meinwald, Wagner–Meerwein and Favorskii reaction), using oxidants based on thallium and iodine, with a focus on recent works published in the period from 2014 to 2024.

## Review

### 1 Recyclization

**Scheme 1:**
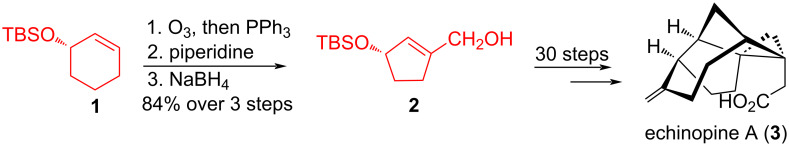
Ozonolysis–cyclization sequence in the synthesis of echinopine A (**3**).

A common method for converting cyclohexene **1** into cyclopentene **2** is the ozonolytic cleavage of the double bond followed by intramolecular aldol condensation of the resulting dialdehyde. This strategy is widely used at the key stage in the formation of a cyclopentane ring in the synthesis of various building blocks, including echinopine A (**3**) [10–12] (Scheme 1).

**Scheme 2:**
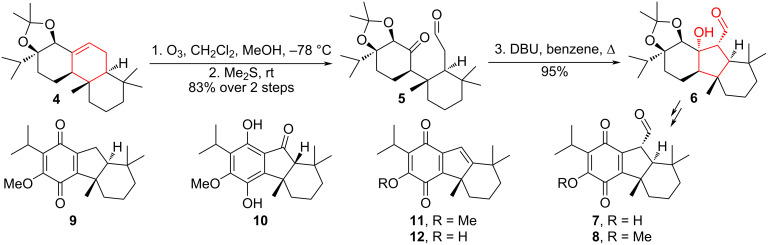
Ozonolysis–cyclization sequence in the synthesis of taiwaniaquinoids **7**–**12**.

The ozonolysis–cyclization sequence was used by Alvarez-Manzaneda et al. as a method for ring contraction of the cyclohexene ring to produce cytostatic taiwaniaquinoids [13–15]. Compound **4**, derived from (–)-abietic acid, was subjected to an oxidative cleavage reaction to produce ketoaldehyde **5**. Subsequent intramolecular aldol cyclization using 1,8-diazobicyclo[5.4.0]undec-7-ene (DBU) in benzene resulted in high yield of β-hydroxyaldehyde **6**. Through a series of synthetic transformations, the target products (−)-taiwaniaquinones A (**7**)*,* F (**8**), G (**9**), and H (**11**), (−)-taiwaniaquinol B (**10**) and (−)-dichroanone (**12**) were obtained from intermediate **6** (Scheme 2).

**Figure 1:**
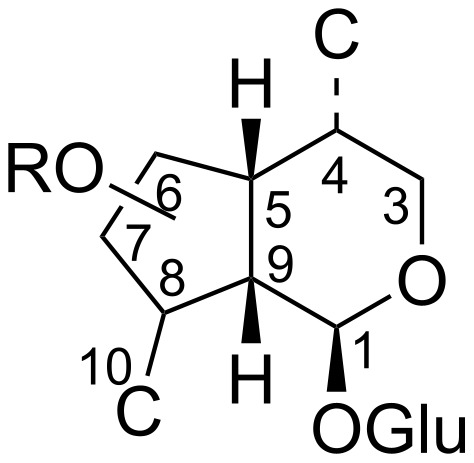
Iridoid skeleton.

An interesting transformation of a cyclohexene fragment into the cyclopentane fragment in Diels–Alder adducts of levoglucosenone (LG) and 1,3-dienes is presented in [16]. Diels–Alder adducts of LG and 1,3-dienes, containing in their structure fragments of cyclohexene and a carbohydrate residue, are attractive objects for investigating the possibility of using them in the synthesis of compounds of iridoid topology. Iridoids belong to the class of terpenoids and are secondary metabolites of plants, as well as multi-tasking objects [5,17–18]. Studies on the pharmacological properties of iridoids have shown their high potential for biological activity, such as anti-inflammatory, antimicrobial, immunomodulatory, neuroprotective, hepatoprotective, hypoglycemic, hypolipidemic, antioxidant, antispasmodic, antitumoral, antiviral, and antiallergic. The breadth of the biological action of these cyclopentanoids is explained to some extent by their structural diversity. Figure 1 shows the general formula of iridoids.

**Scheme 3:**
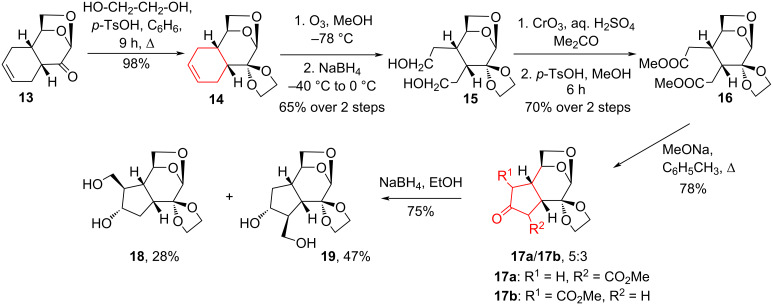
Ozonolysis–cyclization sequence in the synthesis of compounds **17a**,**b**, **18** and **19** with iridoid topologies.

The authors [16] have established regiocontrol of the effect of the 1,6-anhydro bridge on the Dieckmann condensation of dicarboxylic acid diester **16**, obtained in four steps from **13**, the Diels–Alder adduct of LG and 1,3-butadiene (Scheme 3). The ratio of the resulting regioisomers was 5:3 in favor of the 5-methoxycarbonyl derivative **17a**. The synthesized cyclopentane derivatives **17a**,**b**, **18**, and **19** may be useful for the preparation of iridoids and other biologically active cyclopentanoids.

An alternative method for the synthesis of cyclopentane derivatives through oxidative cleavage of the double bond in Diels–Alder adducts of LG with 1,3-butadiene and piperylene (3*E*-penta-1,3-diene), followed by an intramolecular aldol condensation of the resulting dialdehydes was described in [19]. The dicarbonyl compound was synthesized from the Diels–Alder adduct **14** between LG and 1,3-butadiene by two methods – vicinal hydroxylation of the double bond followed by periodate cleavage of the *vic-*diols and ozonolysis of the double bond. Alternatively, Wagner oxidation of the double bond in adduct **14** by treatment with KMnO_4_ in EtOH at 0 °C afforded vicinal diol **20** with a yield of 58%. The periodate cleavage of the latter under the action of NaIO_4_ led to the formation of a labile dialdehyde, which was treated with *t*-BuOK in THF without isolation. As a result of intramolecular aldol condensation, accompanied by the destruction of the main amount of dialdehyde, cyclopentanediol **21** was isolated with a yield of 24%. This synthetic block is promising for the synthesis of gibboside (**22**) [20].

**Scheme 4:**
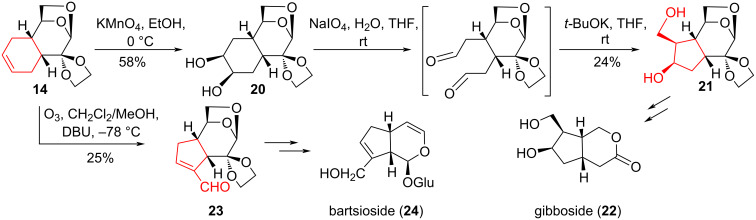
Oxidation–aldol condensation sequence in the synthesis of compounds **21** and **23** with iridoid topology.

Following the second direction, ozonolysis of the double bond in compound **14** and subsequent treatment of the ozonide with DBU resulted in a stable aldehyde **23**. This aldehyde is a product of intramolecular aldol–croton condensation and can be used in the synthesis of bartsioside (**24**) or its analogues [21–22] (Scheme 4).

**Scheme 5:**
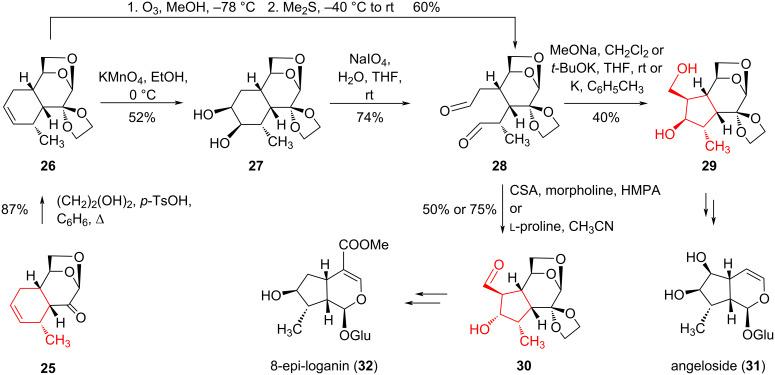
Oxidation–aldol condensation sequence in the synthesis of compounds **29** and **30** with iridoid topology.

The authors [19] performed similar oxidative transformations with Diels–Alder adduct **25** obtained from LG and piperylene, having previously protected its keto group as dioxolane (Scheme 5). Dioxolane **26** was converted to glycol **27** through *vic*-dihydroxylation in moderate yield. Then, dialdehyde **28** was obtained by treatment with NaIO_4_ with a yield of 74%. Subsequent intramolecular aldol condensation of dialdehyde **28** with MeONa or *t-*BuOK as base led to the formation of diol **29** with a yield of 40%, which is interesting for the synthesis of angeloside (**31**) [23].

An alternative preparation of diol **29** involved ozonolysis of the double bond in dioxolane **26** at −78 °C in methanol, followed by reduction of the ozonide with Me_2_S to give dialdehyde **28** and reaction of compound **28** with colloidal potassium in toluene. During aldol condensation in the presence of morpholine-camphorsulfonic acid (CSA) or ʟ-proline, a stable aldehyde **30** was isolated in yields of 50% and 75%, respectively. Decarbonylation and corresponding modifications of the carbohydrate residue in compound **30** resulted in 8-*epi*-loganin (**32**) [24–25]. Thus, as a result of intriguing transformations based on Diels–Alder adducts of LG with butadiene and piperylene, cyclopentane derivatives were obtained, which are promising for the synthesis of natural compounds, particularly iridoids.

**Scheme 6:**
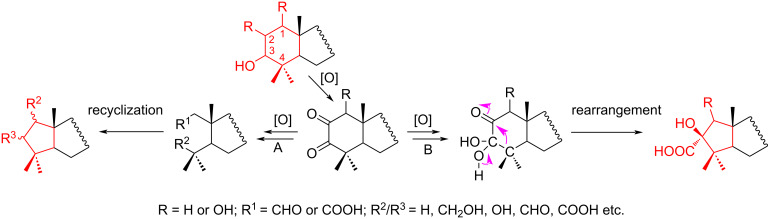
Method for ring contraction in the absence of a double bond in a six-membered ring of triterpenoids.

One of the promising methods for ring contraction in the absence of multiple bonds in the six-membered ring of terpenoids was proposed by Grishko et al. [26] (Scheme 6). The key step of 2,3-fragmentation was the cleavage of an enolized C–C bond at a keto group, followed by an intramolecular aldol reaction (recyclization or rearrangement). This method is applicable to triterpenoids, such as tirucallane, ursane, oleanane and dammarane types (pathway A), as well as for triterpenoids of the ursane and dammarane types (pathway B) [27].

**Scheme 7:**
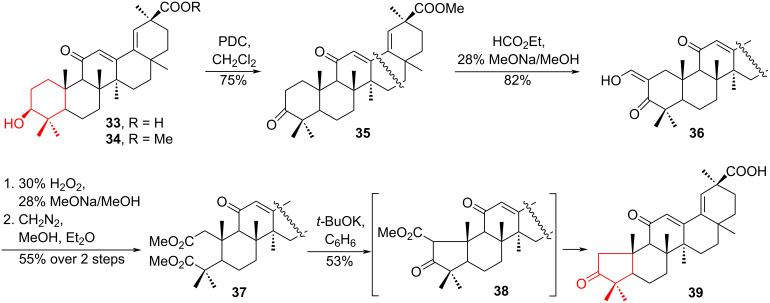
Oxidation–Dieckmann cyclization sequence in the synthesis of a new nortriterpenoid **39**.

In [28], a convenient method for ring contraction is described, which is widely used in the synthesis of triterpenoids. In continuation of studies on skeletal and oxidative transformations of triterpenoids from licorice roots (*Glycyrrhiza glabra L.* and *G. uralensis Fisher*) (leguminosae) [29–31], the authors synthesized a derivative with an altered pentacyclic structure, 2,11-dioxo-A-norolean-12,18(19)-dien-30-oic acid **39**, from pharmaceutical 18,19-dehydroglycyrrhetic acid (18,19-dehydro-GLA) **33**. Compound **33** was converted to its methyl ester **34** and oxidized by pyridinium dichromate (PDC) in CH_2_Cl_2_ to 3-oxo-18,19-dehydro-GLA **35** with a 75% yield. α-Ethylformylation of compound **35** and subsequent oxidation of ketoenol **36** with 30% aqueous H_2_O_2_ in the presence of a 28% MeONa/MeOH solution resulted in the formation of a diacid, which was then converted into dimethyl ester **37** with a yield of 55%. Refluxing ester **37** with an excess of *t-*BuOK in benzene gave acid **39** with a yield of 53% yield. The excess of base (*t-*BuOK) caused simultaneous decarboxylation and hydrolysis of ester **37**, forming the new nortriterpenoid **39** with a pentacyclic ring A (Scheme 7).

**Scheme 8:**
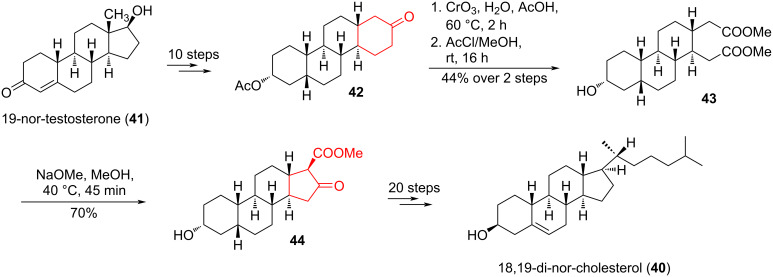
Oxidation–Dieckmann cyclization sequence in the synthesis of 18,19-di-nor-cholesterol (**40**).

In the work of Rath et al. [32], a similar method was proposed for the interconversion of cycles in the synthesis of the demethylated form of cholesterol, 18,19-di-nor-cholesterol (**40**) from commercially available 19-nortestosterone (**41**) (Scheme 8). Molecular modeling of 18,19-di-nor-cholesterol showed that cholesterol’s opposing rough β-face and smooth α-face play crucial roles in cholesterol’s membrane condensing abilities. Specific facial preferences are displayed as cholesterol interacts with different neighboring lipids and transmembrane proteins [33–34]. These biochemical interactions are poorly understood, so the synthesis of a «smoothed» cholesterol analogue provided an opportunity to experimentally measure the significance of the β-face methyl groups.

**Scheme 9:**
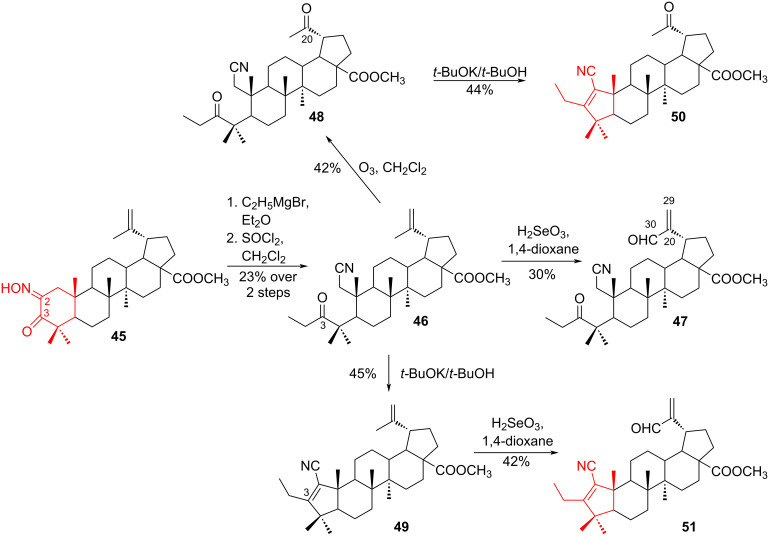
Oxidation–cyclization sequence in the synthesis of 3-ethyl-substituted betulinic acid derivatives **49**–**51**.

The attractive synthesis of 3-ethyl-substituted betulinic acid derivatives with high cytotoxic and anti-inflammatory activity was described by Grishko et al. [35]. The 2,3-secotriterpenic 3-ethyl-3-ketone **46** was obtained in two steps from lupane α-ketoxime **45** by Grignard reduction alkylation, followed by a Beckmann fragmentation of the C2–C3 bond of the intermediate 3-ethyl-substituted hydroxyimino ketone in the SOCl_2_-CH_2_Cl_2_ system. The introduction of a carbonyl substituent into the isopropylidene fragment of ketone **46** was achieved either by allylic oxidation using H_2_SeO_3_-dioxane system to form the C30 aldehyde **47**, or by the ozonolytic cleavage of the double bond between C20 and C29 to produce 20-methyl-3-ethyldiketone **48** [36]. Intramolecular nitrile–anionic cyclization of ketone **46** or diketone **48** under conditions of basic catalysis proceeded via the oxo-nitrile mechanism, with formation of the corresponding Α-pentacyclic 3-ethyl α,β-alkenenitriles **49** and **50** with yields of 44% to 45% (Scheme 9). Cytotoxic screening was performed using the MTT assay with a panel of 7 human tumor cell lines, and showed that the synthesized triterpenoids **47** and **51** showed high cytotoxic activity (IC_50_ 1.38–15.91 μM).

### 2 Rearrangements with 1,2-carbon migration

Rearrangements with 1,2-carbon migration are widely used in the synthesis of natural compounds [37]. These include benzilic acid and semipinacol-type rearrangements, reactions promoted by thallium(III) and iodine(III), and Wolff rearrangement.

#### 2.1 Benzilic acid and semipinacol-type rearrangements

**Scheme 10:**
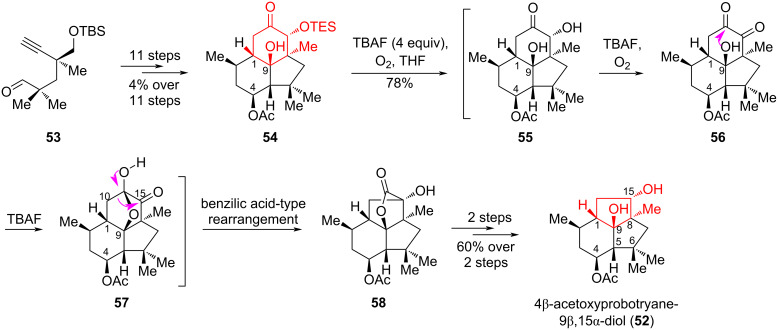
Benzilic acid-type rearrangement in the synthesis of 4β-acetoxyprobotryane-9β,15α-diol (**52**).

The strategy of ring contraction using the benzilic acid-type rearrangement was used by Zhang et al. [38] for the asymmetric synthesis of 4β-acetoxyprobotryane-9β,15α-diol (**52**). This compound contains a sterically compact [6-5-5] tricyclic skeleton, as well as a highly strained *trans-*fused bicyclo[3.3.0]octane ring system and seven contiguous stereocenters. A structurally interesting diol **52** was diastereoselectively obtained using a linear sequence of 14 steps starting from the readily available aldehyde **53** [39] (Scheme 10). The key steps in this synthesis are based on an asymmetric rhodium-catalyzed [4 + 2] cycloaddition reaction [40], followed by a unique benzilic acid-type rearrangement under very mild conditions [41]. A step-by-step mechanism for the benzilic acid-type rearrangement of compound **54** was proposed by the authors [38]. It involves deprotection of the TES group in **54** using tetrabutylammonium fluoride (TBAF) to give **55**. In the presence of O_2_ and TBAF, **55** spontaneously oxidized to **56**. Intramolecular hemiketalization of **56** resulted in the formation of hemiketal **57**, followed by rearrangement to produce lactone **58** with a yield of 78%. The last stage of the synthesis proceeds through a chemo- and diastereoselective reduction of lactone **58**, containing the desired *trans-*fused bicyclo[3.3.0]octane ring system, leading to the target 4β-acetoxyprobotryane-9β,15α-diol (**52**).

**Scheme 11:**
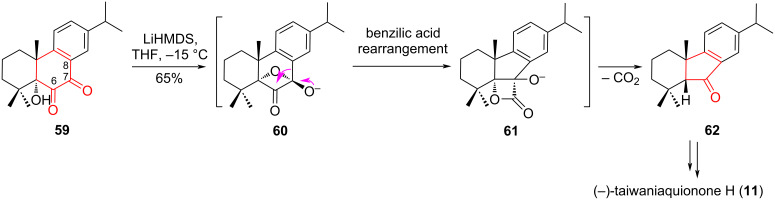
Benzilic acid-type rearrangement in the synthesis of (−)-taiwaniaquinone H **(11**).

An alternative approach to the synthesis of taiwaniaquinoids was applied by Gademann et al. [13,42] for the transition from an abietane structure to a five-membered system using a rearrangement of benzilic acid. The strong base promoted the intramolecular attack of the α-hydroxy group in compound **59** on the keto group at C7, forming synthon **60**. Destruction of this tetrahedral intermediate with migration of the aryl group promoted the formation of intermediate **61**, decarboxylation of which led to the *cis*-substituted product **62**. The resulting ketone **62** was the key synthon in the synthesis of (−)-taiwaniaquinone H (**11**) (Scheme 11).

**Scheme 12:**
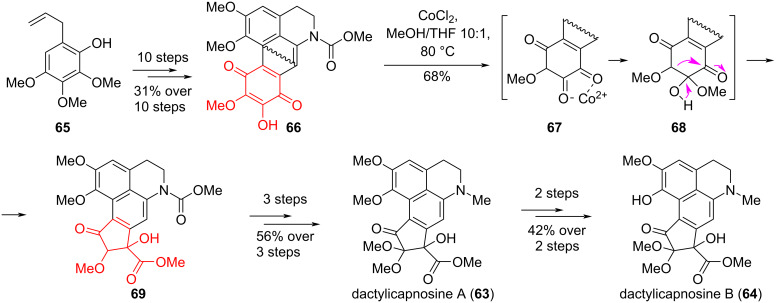
Benzilic acid-type rearrangement in the synthesis of dactylicapnosines A (**63**) and B (**64**).

The technique of regioselective oxidative contraction of a six-membered ring was described in the work by Luo, Zhang and co-workers [43] during their synthesis of the natural alkaloids, dactylicapnosines A (**63**) and B (**64**), which contain a 9,10-seco-7-dehydroaporphinoid skeleton with an unprecedentedly highly oxygenated five-membered D-ring. These compounds exhibit potent anti-inflammatory and analgesic activity both in vitro and in vivo [44]. The synthetic method for the preparation of dactylicapnosines A (**63**) and B (**64**) was based on the known phenol **65** and involved the ring contraction of *p-*quinone **66** through a Co-mediated benzilic acid rearrangement to give cyclopentanone **69** with a 68% yield. The target products **63** and **64** were obtained in 14 and 16 steps with a total yield of 12% and 5%, respectively, from compound **65** (Scheme 12). The presented improved method opens up the possibility of obtaining medically interesting dactylicapnosine-like analogues for detailed study of their biological activity.

**Scheme 13:**
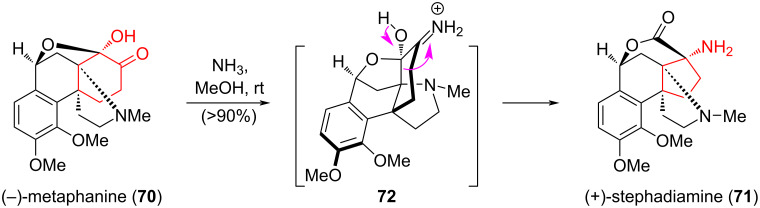
Aza-benzilic acid-type rearrangement in the synthesis of (+)-stephadiamine (**71**).

Matoba et al. [45] reported the first enantioselective synthesis of the hasubanane alkaloid (−)-metaphanine (**70**) and the norhasubanane alkaloid (+)-stephadiamine (**71**) using a cyclohexane ring contraction through aza-benzilic acid-type rearrangement (Scheme 13). Hasubanan-type alkaloids exhibit a wide range of biological activities, including antiviral, antimicrobial, and cytotoxic activities [46], while the biological activities and synthesis of structurally attractive norhasubanan-type alkaloids have been little studied, and are of interest to synthetic chemists. Thus, the interaction of (−)-metaphanine (**70**) with ammonia in CH_3_OH at room temperature led to the in situ formation of imine **72**, which, as a result of an intramolecular aza-benzilic acid-type rearrangement, was converted in more than 90% yield to (+)-stephadiamine (**71**). The authors noted that the identification of the structure of (+)-stephadiamine (**71**) was carried out without purification of the reaction mixture due to its instability on a silica gel column under acidic or basic conditions.

**Scheme 14:**
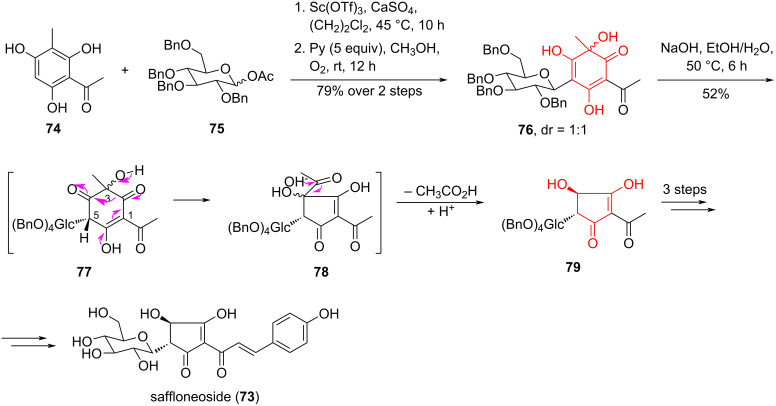
α-Ketol rearrangement in the synthesis of saffloneoside (**73**).

Zhang et al. [47] proposed an efficient seven-step synthesis of the new, structurally interesting *p*-hydroxycinnamylcyclopentenone *C*-glucoside saffloneoside (**73**), isolated from the inflorescences of сarthamus tinctorius. *Carthamus tinctorius L.* is a medicinal plant that can be used to treat ischemic stroke [48]. In their study [47] they reported a stereospecific reduction of an acyloin ring controlled by the chirality of a glucose fragment at position C5, and suggested a possible mechanism for hydrogen migration during the conversion of C5 (sp^2^) to C5 (sp^3^). The key 3,4,6-trihydroxycyclohexadienone **76** was obtained by a sequence of Friedel–Crafts-type glucosylation reactions of 2,4,6-trihydroxy-3-methylacetophenone (**74**) with glucosyl acetate **75** in the of presence Sc(OTf)_3_ (0.4 equiv) as a Lewis acid promoter and oxidative dearomatization of the *C-*glucoside precursor by O_2_ in the presence of pyridine in CH_3_OH. The key step in the acyloin ring contraction of compound **76** was the stereospecific formation of the cyclopentenone-containing intermediate **78**, by α-ketol rearrangement under the action of NaOH solution. After cleavage of the acyl moiety in synthon **78** under basic conditions, diastereomerically pure cyclopentenone **79** was obtained with 52% yield (Scheme 14). The authors [47] provided convincing evidence that the acyloin ring contraction is stereospecific, by replacing the benzil ᴅ-glucose in compound **76** at the C5 atom with an achiral prenyl group.

**Scheme 15:**
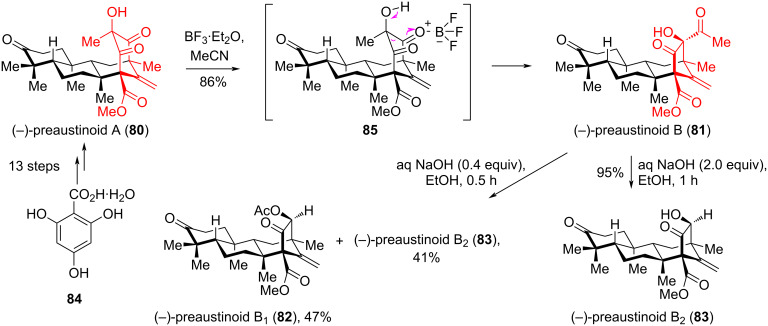
Conversion of (−)-preaustinoid A (**80**) to (−)-preaustinoid B (**81**) via α-ketol rearrangement.

In [49], Zhang and co-workers carried out the first enantioselective synthesis of fungal meroterpenoids ((−)-preaustinoids A (**80**), B (**81**), B_1_ (**82**), B_2_ (**83**)) from commercially available 2,4,6-trihydroxybenzoic acid hydrate (**84**) using a cyclohexanone ring contraction strategy – α-ketol rearrangement. Fungal meroterpenoids derived from a simple aromatic polyketide, 3,5-dimethylorcellinic acid (DMOA), are a large series of hybrid natural products with a huge structural diversity and impressive biological activity [50]. Attempts by the authors to use acidic (TiCl_4_, TMSI, SnCl_4_), basic (NaOMe, *t*-BuONa, Et_3_N, DBU, DMAP) and thermal conditions (PhMe, 110 °C) failed to give the desired rearrangement product. However, treatment of (−)-preaustinoid A (**80**) with BF_3_·Et_2_O in MeCN led to an α-ketol rearrangement affording (−)-preaustinoid B (**81**) as the sole product in 86% yield. Presumably, this reaction proceeded through intermediate **85**. Subsequently, the deacylation of the product **81** with 2.0 equivalents of aqueous NaOH in EtOH, afforded (−)-preaustinoid B_2_ (**83**) with 95% yield. Using 0.4 equivalents of aqueous NaOH, a mixture of (−)-preaustinoid B1 (**82**) and (−)-preaustinoid (**83**) was obtained (Scheme 15).

**Scheme 16:**
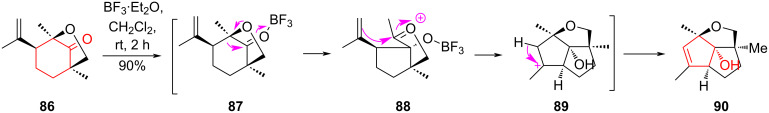
α-Ketol rearrangement in the synthesis of 2,8-oxymethano-bridged diquinane **90**.

Nagaraju and Prasad used the α-ketol rearrangement mediated by Lewis acids to carry out the ring contraction reaction on simple cyclohexanones to synthesize functionalized di- and triquinanes [51]. Treatment of 6-oxabicyclo[3.2.1]octan-8-one **86** with BF_3_·Et_2_O (2 equiv) promoted the activation of the carbonyl group in **87** by the Lewis acid, leading to the formation of an oxocarbenium ion **88**. Next, ion **88** reacted with the isopropenyl group, forming intermediate **89**, which was then converted into the desired 2,8-oxymethano-bridged diquinane **90** in 90% yield (Scheme 16).

In the enantioselective synthesis of sesquiterpenoids (+)-cuparene (**91**) and (+)-tochuinyl acetate (**92**), Xie and co-workers [52] achieved high regioselectivity and stereospecific construction of contiguous all-carbon quaternary centers through an oxidative ring contraction of cyclic α-formyl ketones by the action of H_2_O_2_. This reaction is easy to perform, environmentally friendly, and does not require expensive catalysts.

**Scheme 17:**
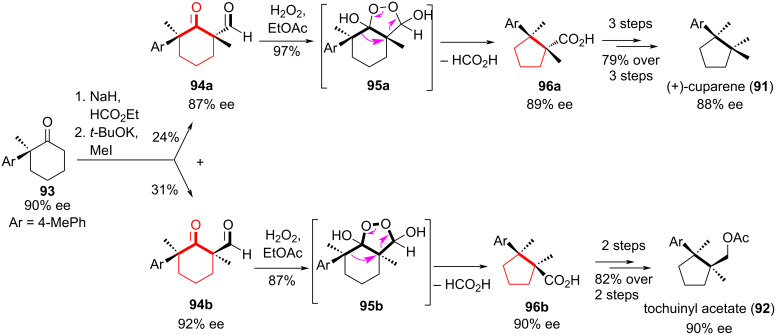
Oxidative ring contraction during the synthesis of (+)-cuparene (**9**1) and (+)-tochuinylacetate (**92**).

Formylation of enantioenriched hexanone **93** (90% ee) with NaH/HCO_2_Et, followed by methylation produced two separable isomers, **94a** and **94b**, in yields of 24% and 31%, respectively. Treatment of these isomers with H_2_O_2_ smoothly delivered the carboxylic acids **96a** and **96b** stereospecifically with excellent regioselectivity (>99:1) and complete preservation of the stereochemistry at both quaternary carbon centers (Scheme 17). Through a series of synthetic transformations, the target products (+)-cuparene (**91**) and (+)-tochuinylacetate (**92**) were synthesized from acids **96a** and **96b** with high regioselectivity. In [53], the mechanism and factors controlling the selectivity of the oxidative ring contraction in cyclic α-formyl ketones under the action of H_2_O_2_ were studied in detail.

**Scheme 18:**
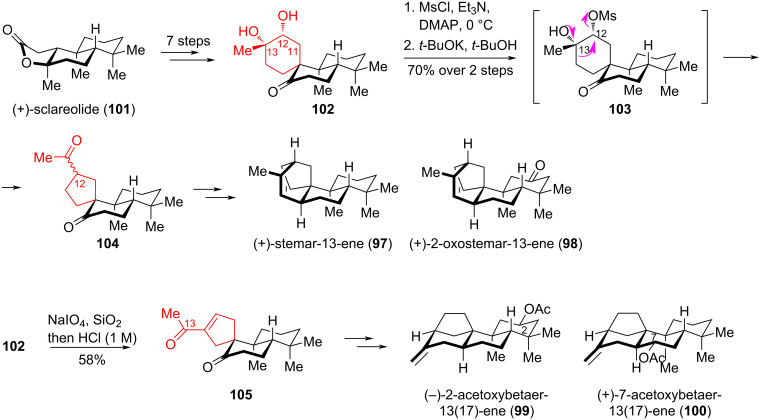
Semipinacol rearrangement in the synthesis of diterpenoids **97**–**100**.

Due to their complex architecture and diverse biological activities, including antiviral, antitumor, antimalarial, and antifungal activity [54], stemarenes **97**, **98** and betaerene **99**, **100** diterpenoids attracted the attention of synthetic chemists. In [55], Chen et al. described the elegant synthesis of these tetracyclic diterpenoids using a semipinacol rearrangement as a ring contraction technique (Scheme 18). The authors used commercially available (+)-sclareolide (**101**) as starting compound, from which key alcohol **102** was synthesized in 7 steps. Selective mesylation of the secondary alcohol in **102** followed by semipinacol rearrangement catalyzed by *t-*BuOK afforded diketone **104** as a pair of epimers at C12 in a 1:1 ratio, with 70% overall yield. Cleavage of diol **102** with silica-supported NaIO_4_ generated an unstable tricarbonyl intermediate, which without puriﬁcation was rapidly treated with 1 M HCl to produce enone **105** with a yield of 58%. Through a series of synthetic transformations from the obtained diketones **104** and **105**, the target diterpenoids **97**–**100** containing pseudoenantiomeric bridged C/D rings were obtained.

**Scheme 19:**
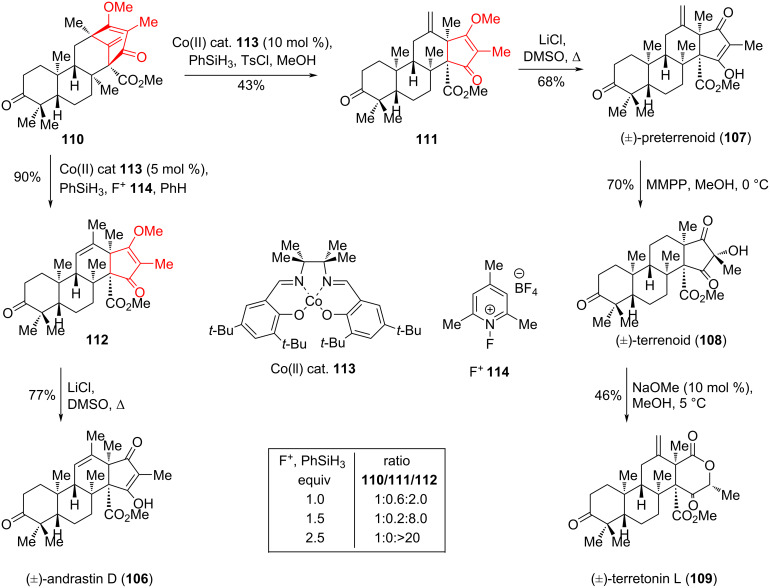
Co-catalyzed homoallyl-type rearrangement in the syntheses of meroterpenes **106**–**109**.

Andrastins and terretonins represent a large subset of 3,5-dimethylorcellinic acid (DMOA)-derived members with interesting biological profiles and unique carbocyclic frameworks. In 2017, Newhouse, Maimone and colleagues [56] described the first total syntheses of racemic meroterpenes (±)-andrastin D (**106**), (±)-preterrenoid (**107**), (±)-terrenoid (**108**), and (±)-terretonin L (**109**) using a Co-catalyzed homoallyl-type rearrangement/hydrogen atom transfer (HAT) as ring contraction strategy. Radical hydrochlorination of olefin **110** [57] using Co(II) catalyst **113**, in the presence of PhSiH_3_, TsCl in MeOH resulted in the formation of a cyclopentenone derivative **111** with a yield of 43% and a small amount (<5%) of cyclopentenone derivative **112**. The remaining mass balance consisted mainly of the recovered starting material. Demethylation of **111** led to (±)-preterrenoid (**107**) with 68% yield, which was subjected to stereoselective oxidation with magnesium monoperoxyphthalate (MMPP) to give (±)-terrenoid (**108**) as a single diastereomer. Subsequent treatment of the key precursor **108** with catalytic amounts of NaOMe in MeOH resulted in (±)-terretonin L (**109**) as the main product with a yield of 46%. Radical hydrochlorination of olefin **110** under more powerful oxidizing conditions (Co(II) catalyst **113**, *N-*fluoropyridinium salt (F^+^) **114**), resulted in the formation of cyclopentenone derivative **112** with a yield of 90%. Demethylation of the latter led to (±)-andrastin D (**106**) at a yield of 77% (Scheme 19).

#### 2.2 Ring-contraction reactions promoted by thallium(III) and iodine(III)

**Scheme 20:**
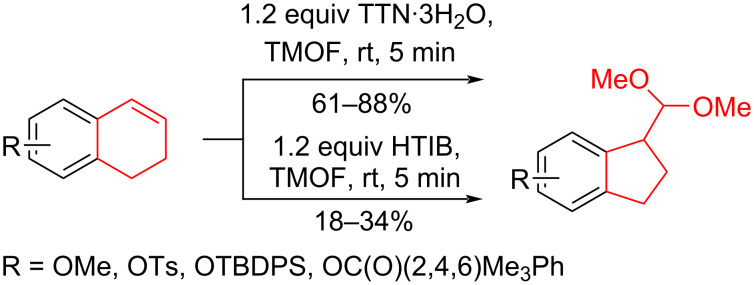
Ring contraction reaction promoted by TTN·3H_2_O and HTIB in the synthesis of indanes.

Silva et al. [58–59] described a universal and efficient strategy for the synthesis of indanes using a ring contraction reaction mediated by iodine(III) and thallium(III) (Scheme 20). Indanes (benzocyclopentanes) and their derivatives are widely used in materials science and nanotechnology and have important biological properties such as anti-allergic, antitumor, anticonvulsive, antihypercholesteremic, herbicidal, fungicidal and antimicrobial activity [60]. In [58], a comparative study of two effective and practical oxidizing agents, hydroxy(tosyloxy)iodobenzene (HTIB) and thallium nitrate trihydrate (TTN·3H_2_O), was presented in the ring contraction reaction of protected 1,2-dihydronaphthalenes using trimethylorthoformate (TMOF). In addition, various protective groups were introduced to substrates, aiming to study their tolerance for both oxidizing agents (Table 1). The yields of the ring contraction products (indanes) **116a**–**g** ranged from 61 to 88% when the reactions were carried out with TTN·3H_2_O in TMOF. When HTIB was used in TMOF, the yields were significantly lower, ranging from 18% to 34% (Scheme 20). Analysis of literature data [58] indicated that the thallium(III) salt is a much better oxidizing agent than iodine(III) for indane syntheses. Similarly, the TTN-mediated reactions are less prone to form addition by-products compared to the HTIB-mediated reactions of 1,2-dihydronaphthalenes.

**Table 1:** Oxidative contraction of 1,2-dihydronaphthalene derivatives **115a**–**g** under the action of TTN·3H_2_O and HTIB during the synthesis of indanes **116a**–**g**.

Entry	Substrate	Acetal (indane)	Indane yield under the action of	

			HTIB in TMOF	TTN·3H_2_O in TMOF

1	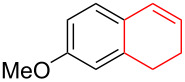 **115a**	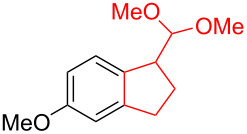 **116a**	29%	88%
2	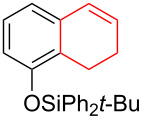 **115b**	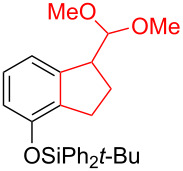 **116b**	28%	74%
3	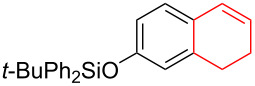 **115c**	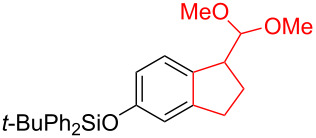 **116c**	34%	79%
4	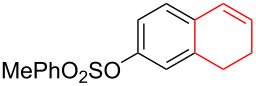 **115d**	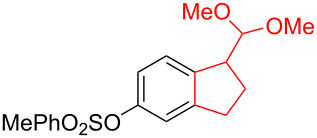 **116d**	29%	69%
5	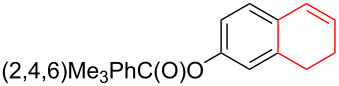 **115e**	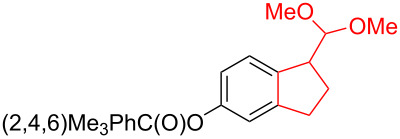 **116e**	31%	61%
6	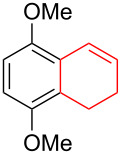 **115f**	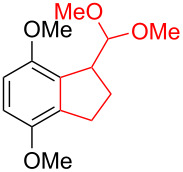 **116f**	18%	72%
7	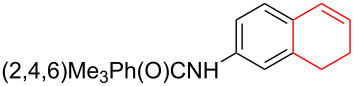 **115g**	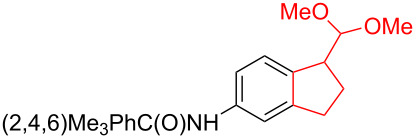 **116g**	32%	68%

**Scheme 21:**
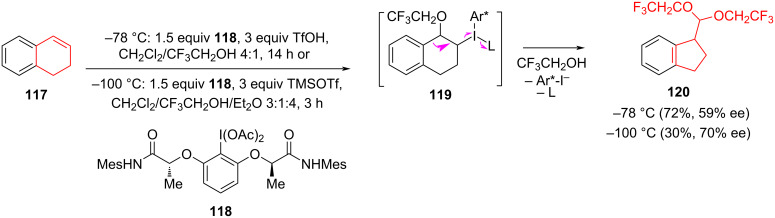
Rearrangement involving a hypervalent iodine compound in the synthesis of derivative **120**.

Silva et al. have also developed an effective method for the stereoselective oxidation of tetralone derivatives using chiral hypervalent iodine reagents [61–62]. Hypervalent iodine compounds are widely used in organic synthesis as selective oxidants and enantiomerically pure reagents. In terms of reactivity, they are environmentally sustainable alternatives to heavy metals. The advantage of hypervalent catalytic systems based on iodine lies in their reusability [63]. The oxidative contraction of the tetralone cycle **117** depended on the stoichiometric amounts of iodine(III)-based chiral reagent **118** using trifluoromethanesulfonic acid (TfOH) or TMSOTf as Lewis acids. The authors suggested that the reaction proceeded via the formation of an intermediate **119** followed by reaction with an alcohol nucleophile and a 1,2-carbon migration to produce a cyclopentane derivative **120** with a yield of 30% and a selectivity of 70% at −100 °C (Scheme 21).

#### 2.3 Wolff rearrangement

The Wolff rearrangement is the transformation of α-diazoketones into acids or their derivatives through heating, catalysis, or UV irradiation in the presence of water, alcohols, ammonia, amines, etc.

**Scheme 22:**
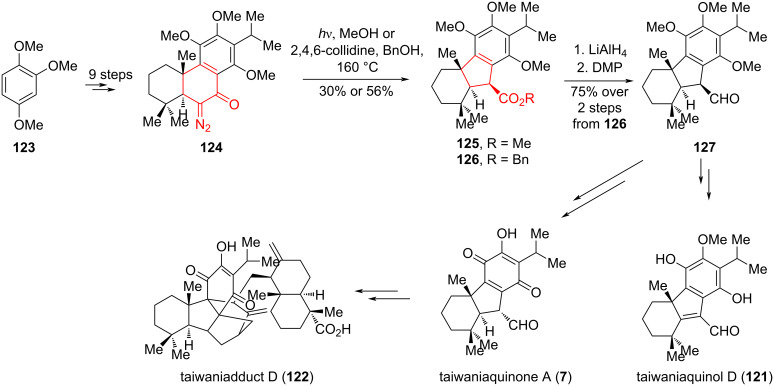
Wolff rearrangement in the synthesis of taiwaniaquinones A (**7**), F (**8**), taiwaniaquinols B (**10**), D (**121**), and taiwaniadducts B, C, and D **122**.

The Wolff rearrangement is a convenient method for forming a cyclopentane ring in the synthesis of racemic forms of taiwaniaquinones A (**7**), F (**8**), and taiwaniaquinols B (**10**), D (**121**) [64] and taiwaniadducts B, C and D **122** [65], proposed by Deng et al. (Scheme 22). Taiwaniaquinoids are a class of terpenoids with an unusual 6,5,6-abeoabietane scaffold and impressive antitumor activity. α-Diazoketone **124** was synthesized from commercially available 1,2,4-trimethoxybenzene (**123**) [64] by a series of synthetic transformations, and converted by Wolff rearrangement to esters **125** and **126** with a *trans-*fused 6,5,6-ring system. Irritation of a solution of **124** in CH_3_OH using a medium-pressure Hg lamp resulted in the formation of methyl ester **125** as a single diastereomer with a yield of 30%. Due to the decrease in the efficiency of this reaction at higher loads (>50 mg), the thermal conditions for the Wolff rearrangement were tested (BnOH, 2,4,6-collidine, 160 °C). The desired benzyl ester **126** was isolated as single diastereomer with a yield of 56% yield. Reduction of **126** with LiAlH_4_ followed by oxidation with Dess–Martin periodinane (DMP) led to the synthesis of a key aldehyde **127**, with a yield of 75% in two steps. The target products were synthesized through a series of synthetic transformations starting with **127**.

**Scheme 23:**
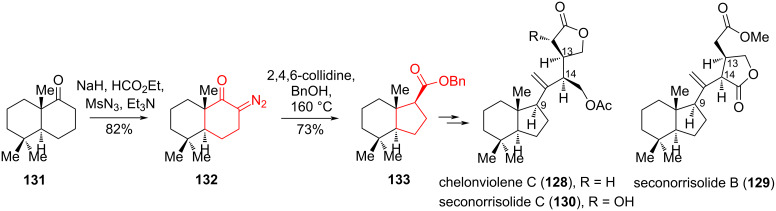
Wolff rearrangement in the synthesis of cheloviolene C (**128**), seconorrisolide B (**129**), and seconorrisolide C (**130**).

In [66], it was shown that the Wolff rearrangement is an effective method for obtaining a transhydrindane system in norrisolide-type rearranged spongian diterpenes cheloviolene C (**128**), seconorrisolide B (**129**), and seconorrisolide C (**130**) from Wieland–Miescher ketone derivative **131**. The spongian diterpenes are a large family of marine natural products that have pronounced effects on the Golgi apparatus [67]. The introduction of the diazo group into ketone **131** was achieved under standard conditions through an α-formyl intermediate. The Wolff rearrangement was carried out by heating **132** in collidine and BnOH to obtain *trans-*hydrindane **133** as a single diastereomer with a yield of 73% (Scheme 23).

**Scheme 24:**
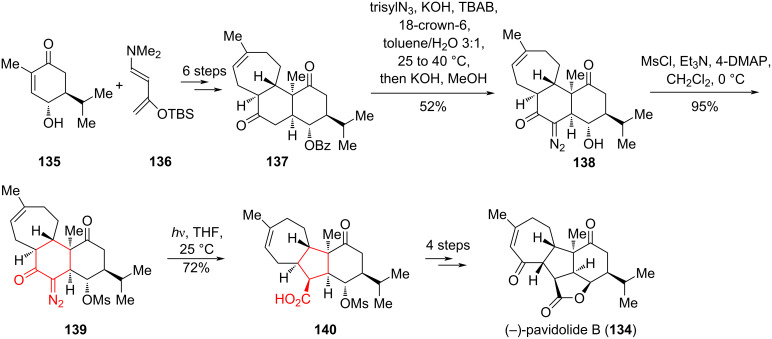
Wolff rearrangement in the synthesis of (−)-pavidolide B (**134**).

In the work of Ding et al. [68], an elegant synthesis of (−)-pavidolide B (**134**) was described using the Wolff rearrangement. In 2012, Lin and co-workers [69] isolated the membrane diterpenoid (−)-**134** from the marine soft coral *Sinularia pavida*. The study showed that (−)-**134** exhibits highly selective inhibition against the human promyelocytic leukemia cell line HL-60 with an IC_50_ of 2.7 μg/mL. Structurally, (−)-**134** contains a challenging [6-5-5-7]-tetracyclic ring system embedded within a fully functionalized cyclopentane, as well as seven contiguous stereogenic centers. In 2017, (−)-pavidolide B (**134**) was prepared in 13 steps from the known chiral alcohol **135**, which in turn was synthesized from commercially available (+)-carvone in two steps [70]. The intermediate tricyclic ketone **137** was converted to the corresponding α-diazoketone through treatment with 2,4,6-triisopropylbenzenesulfonyl azide (trisylN_3_), KOH, tetrabutylammonium bromide (TBAB), and 18-crown-6. Concurrent cleavage of the benzoyl group under basic conditions led to the isolation of alcohol **138** with a yield of 52%. The Wolff rearrangement was carried out upon irradiation of a solution of mesylate **139** in THF at 25 °C with a medium-pressure Hg lamp for 1 hour. The ring contraction proceeded diastereoselectively and the desired acid **140** was obtained as a single diastereomer with a yield of 72%. A subsequent series of synthetic transformations on acid **140** resulted in the target (−)-pavidolide B (**134**) (Scheme 24).

**Scheme 25:**
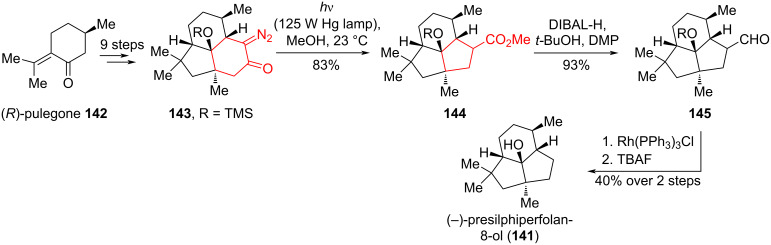
Wolff rearrangement in the synthesis of presilphiperfolan-8-ol (**141**).

Hu and Snyder [71] carried out the first enantioselective synthesis of the highly strained natural product (−)-presilphiperfolan-8-ol (**141**) in 13 steps from commercially available (*R*)-pulegone (**142**) using a ring contraction strategy based on the Wolff rearrangement. By irradiating diazo ketone **143** for 20 minutes at 23 °C in MeOH, the desired product **144** was obtained with a yield of 83%. The synthesis of (−)-presilphiperfolan-8-ol (**141**) was completed by converting the exocyclic methyl ester **144** to an aldehyde**145** using a one-pot DIBAL-H/Dess–Martin procedure followed by the removal of TMS protecting group using a stoichiometric Wilkinson’s catalyst (Scheme 25). The authors noted that, in the final stages of reduction and oxidation, yields were lower when **145** was obtained (70% vs 93%). At the same time, the reaction with (PPh_3_)_3_Cl in small amounts (≈15 mg) proceeded smoothly.

### 3 Photochemical rearrangements

Photochemical reactions are a powerful tool for quickly accessing a variety of structural and stereochemical compounds from relatively simple building blocks [72]. The main feature of light-induced transformations is the involvement of electronically excited states that occur during photon absorption, leading to the formation of reactive intermediates causing significant changes in chemical compound reactivity. As a result, light energy can be used to synthesize complex and unique compounds that cannot be produced by thermal reactions, making photochemical transformations preferable to classical ones, especially when considering the concept of «green chemistry» [73]. Therefore, photochemical rearrangements have great theoretical and practical importance when considering various transformations including the transformation of hexanes into cyclopentanes.

**Scheme 26:**
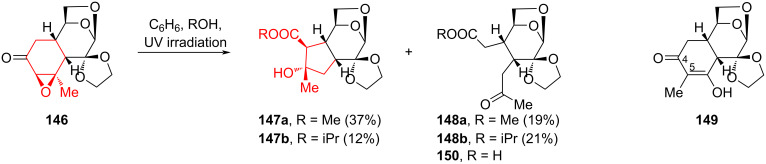
Photochemical rearrangement in the synthesis of cyclopentane derivatives **147a**,**b**.

An interesting photochemical rearrangement of 5,6-epoxy derivatives of the Diels–Alder adduct of LG and piperylene was described in reference [74]. This reaction leads to the formation of cyclopentane derivatives, promising for the synthesis of iridoids and compounds, that stimulate the formation of receptors that prevent platelet aggregation [75]. The derivatives of the Diels–Alder adduct of LG and piperylene, keto epoxide **146** and its analogue **152** lacking the spiro-fused ethylenedioxy fragment (see below) are attractive substrates for studying the possibility of a photochemically induced contraction of the cyclohexane ring. Photoirradiation of keto oxirane **146** [76] in benzene containing 1% methanol afforded cyclopentane annulated derivative **147a** along with keto ester **148a**, a product of cyclohexane ring opening (Scheme 26). A decrease in the methanol content of the mixture to 0.5% was accompanied by a slowdown in the process and the appearance of keto enol **149** in solution. Replacing methanol with isopropanol under these conditions resulted in the formation of isopropyl esters **147b** and **148b**. The authors of [74] suggested that keto enol **149** was the intermediate from which the cyclopentane derivative **147** and fragmentation products **148** and **150** were formed. Irradiation of a solution of keto enol **149** in benzene containing 1% methanol resulted in the formation of the proposed products **147a** and **148a**. The authors of [74] believe that methyl ketones **148a**,**b** were obtained as a result of breaking the C4–C5 bond, and cyclopentanes **147a**,**b** were formed from methyl ketones through intramolecular aldol condensation.

**Scheme 27:**
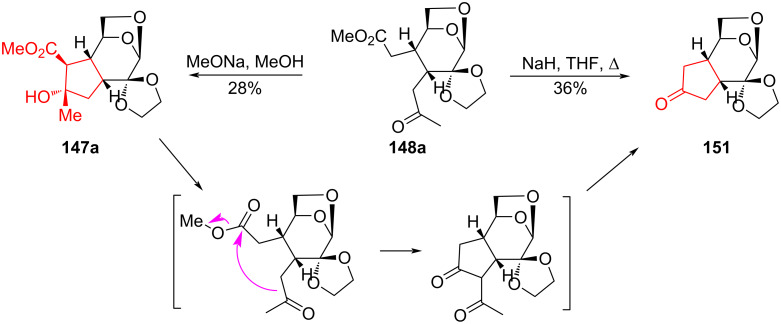
Synthesis of cyclopentane derivatives **147a** and **151**.

Treatment of keto ester **148a** with NaH led to a complex mixture, from which cyclopentanone annulated derivative **151** was isolated with a yield of 36%. Performing the reaction under milder conditions (MeONa/MeOH) yielded cyclopentane derivative **147a** with a yield of 28%. The latter compound, when boiled in THF solution in the presence of NaH produced cyclopentanone derivative **151** (Scheme 27). According to the authors, this transformation proceeded through stages of retro-Claisen condensation, intramolecular aldol-type condensation, and retro-Claisen deacetylation [77].

**Scheme 28:**
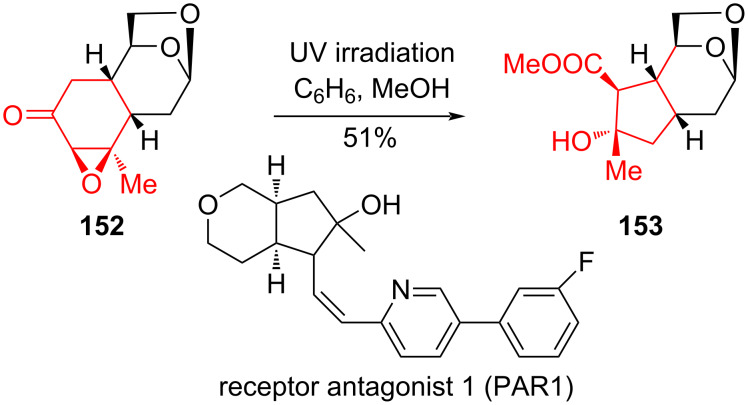
Photochemical rearrangement in the synthesis of cyclopentane derivative **153**.

Photolysis of compound **152** proceeded more efficiently in C_6_H_6_/MeOH (1%) resulting in cyclopentanone derivative **153** with a yield of 51% (Scheme 28).

As a result of the photolysis of 5,6-epoxy ketones **146** and **152**, valuable derivatives of cyclopentane, **147a**,**b**, **151**, and **153** were obtained.

**Scheme 29:**
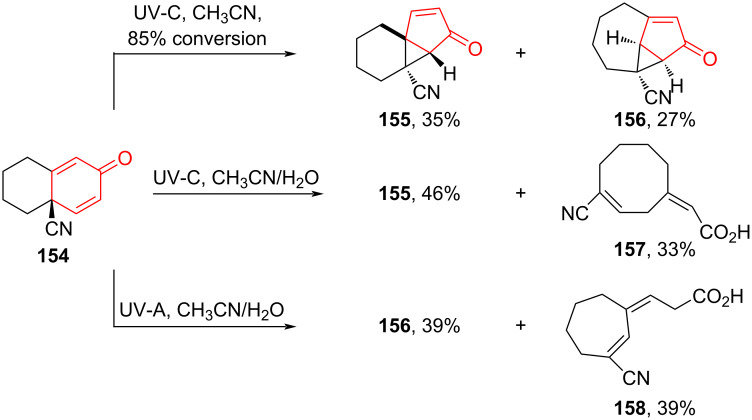
Photochemical rearrangement in the synthesis of tricyclic ketones **155**, **156**.

Photochemistry is an important tool in the synthesis of natural and synthetic biologically active substances based on polycyclic compounds [78–82]. The work of Davis, Derksen and co-workers [83] presents interesting results on the use of UV light to study the reactivity of bicyclic divinyl ketones and the dependence of photochemical reaction products on wavelength. Under anhydrous conditions, irradiation of divinyl ketone **154** with UV-C (200–280 nm) light yielded approximately equal amounts of products **155** and **156** (1.25:1 after 85% conversion). In the presence of water (CH_3_CN/H_2_O 10:1), a crystalline cyclooctene compound **157** was obtained by irradiation of **154**. However, compound **156** was not detected. When irradiating **154** with UV-A (315–400 nm) in the presence of water (CH_3_CN/H_2_O 10:1), cycloheptene compound **158** was obtained, but compound **155** could not be detected. These results are consistent with the selective conversion of ketone **155** to acid **158** by UV-A irradiation and the selective transformation of ketone **156** to acid **157** by UV-C, which occurred only in the presence of water (Scheme 29). In the work [83], it is shown that a vinylogous Norrish Type II cascade reaction can occur for unsaturated ketones substituted with cyclopropanes.

**Scheme 30:**
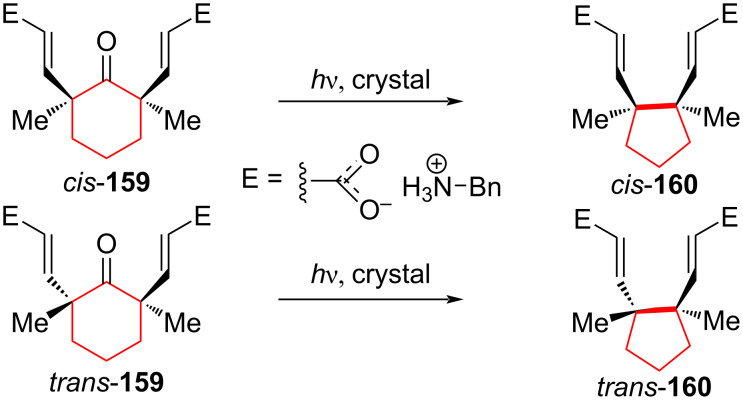
Photochemical rearrangement in the synthesis of *cis*/*trans* salts **160**.

Interesting results of photochemical ring contraction were described by Dotson et al. [84] for *cis*/*trans* salts **159** (Scheme 30). The authors noted that photochemical experiments carried out in benzene solutions with benzylic esters *cis*-**159** or *trans*-**159** gave complex product mixtures. To achieve higher conversion, a photochemical reaction was carried out in the crystalline phase. Benzyl esters were cleaved, and the resulting carboxylic acids were converted into corresponding benzylammonium salts by addition of two equivalents of benzylamine. Subsequent irradiation of these salts in the form of a crystalline suspension in hexane led to ring-contraction products with a 95% yield.

**Figure 2:**
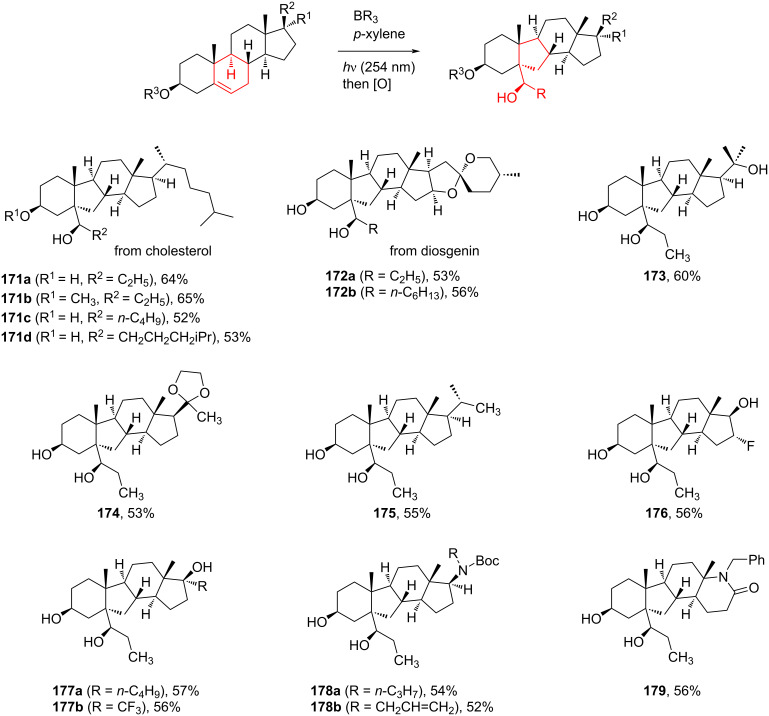
Scope of the photoinduced carboborative ring contraction of steroids. Reaction conditions: steroid derivative (0.23–0.5 mmol), trialkylborane (0.3–0.6 mmol), EtOH or THF (2–4 mL), *p*-xylene (0.5–1 mL), UV (254 nm), then H_2_O_2_, NaOH.

Jin et al. [85] reported an efficient photoinduced carboborative ring contraction of monounsaturated six-membered carbo- and heterocycles, allowing the regio- and stereoselective synthesis of functionalized cyclopentanes at gram scales. This method leads to the formation of compounds with multiple stereocenters, including contiguous quaternary carbons. As a result, the carboborative ring contraction reaction is conveniently used for structural modification of natural products containing a cyclohexene ring and in the synthesis of new compounds to increase their activity, metabolic stability, and target specificity [86]. Impressive results were obtained by testing the photoinduced carboborative ring contraction reaction of terpenoids and steroids. The reactions proceeded with high regio- and stereoselectivity, leading to the formation of substituted cyclopentanes **162**, **164a**,**b**, **166**, **168**, **170**, derivatives of cholesterol **171a**,**b**,**c**,**d**, diosgenin **172a**,**b**, pregnenolone **173–175**, dehydroepiandrosterone **176**, **177a**,**b**, and azasteroids **178a**,**b**, **179**, in the form of single diastereomers (Table 2 and Figure 2) [85].

**Table 2:** Scope of the photoinduced carboborative ring contraction of terpenoids.

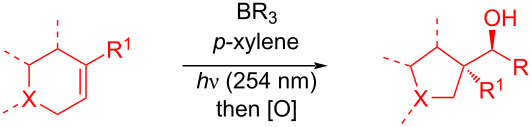			

Terpenoid	Product, yield, dr	Terpenoid	Product, yield, dr

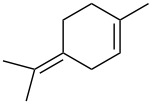 terpinolene **161**	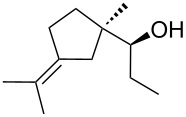 **162**, 91%, >20:1	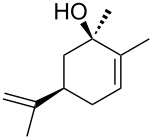 **167**	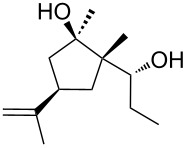 **168**, 60%
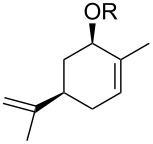 (*R*,*R*)-carveol **163a**, R = H **163b**, R = TBS	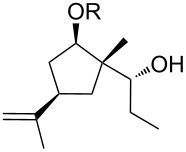 **164a**, 86%, 10:1 **164b**, 63%, >15:1	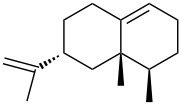 valencene **169**	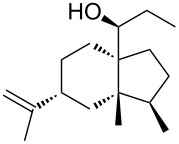 **170**, 74%
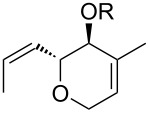 nerol oxide **165**	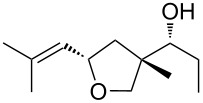 **166**, 52%		

^a^Reaction conditions: terpenoid (1 mmol), trialkyl borane (1–1.5 mmol), EtOH (5 mL), *p*-xylene (2 mL), UV (254 nm), then H_2_O_2_, NaOH, or Na_2_CO_3_·1.5H_2_O_2_.

**Scheme 31:**
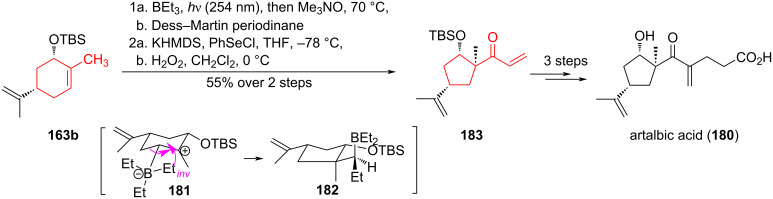
Photoinduced carboborative ring contraction in the synthesis of artalbic acid (**180**).

In the five-step synthesis of artalbic acid (**180**), the authors [85] used a photoinduced carboborative ring contraction of (*S,S*)-carveol-derived TBS ether **163b** with triethylborane (Scheme 31). This reaction proceeded with isomerization and formation of the intermediate carbocation **181**, which underwent 1,2-carbon migration to form the cyclopentane product **182**. A sequence of oxidations of borane **182** with trimethylamine *N*-oxide and Dess–Martin periodinane, α-selenylation, and hydrogen peroxide-induced selenoxide elimination led to unsaturated ketone **183**, from which artalbic acid (**180**) was obtained through a series of chemical transformations.

**Scheme 32:**
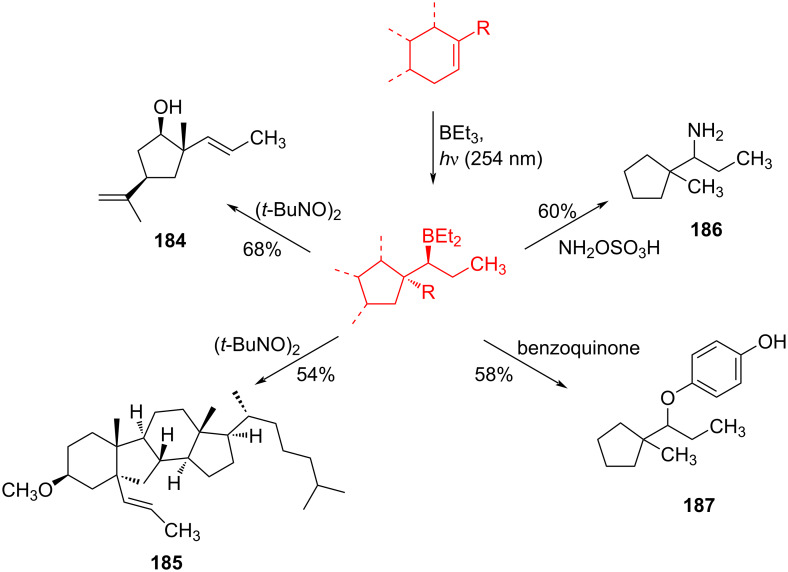
Synthetic versatility of the photoinduced carboborative ring contraction.

The synthetic versatility of the photoinduced carboborative ring contraction is demonstrated by the example of converting organoborane intermediates into alcohols, amines, and *E-*alkenes (Scheme 32).

### 4 Meinwald rearrangements of epoxides

**Scheme 33:**
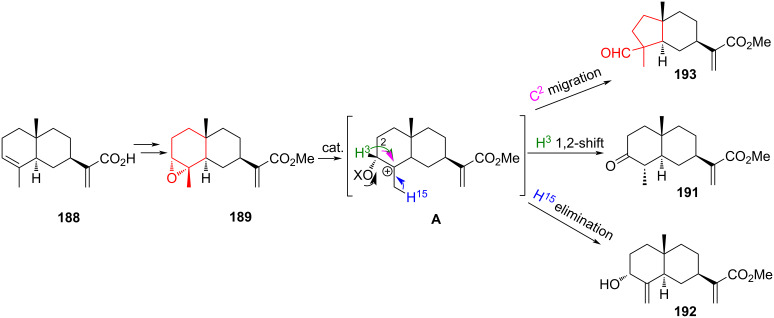
Methods of disclosure of epoxide **189**.

The rearrangements of oxiranes annelated to cyclohexane occupy an important place in the synthesis of cyclopentanes due to the stereospecificity of the reactions. Lewis acids usually initiate this transformation, and the reaction is most efficient when there are electron-accepting groups present. Sometimes, the rearrangement of epoxides into cyclopentane is accompanied by competing reactions – the formation of cyclohexanone/ols – which are products of epoxide ring opening. It should be noted that the position of the epoxy ring in the 6-membered ring has a major influence on the composition of reaction products. Catalysts only affect the yield of the reaction. A striking confirmation of this is the work of Berteina-Raboin and co-workers [87], which describes the acid-catalyzed rearrangement of epoxides **189** and **190** derived from sesquiterpernic α-isocostic acid **188** under the action of various Lewis acids: BF_3_·Et_2_O, InCl_3_, TfOH, ZnBr_2_, Bi(OTf)_3_, *p-*TSA, or TFA. Under the action of the listed acids, the epoxy ring in compound **189** was opened to form three possible products: ketone **191**, olefin **192**, and cyclopentane **193** (Scheme 33). At the first stage, the epoxy ring was opened to form intermediate **A**. According to the authors [87], there are three possible ways of carbocation stabilization. The 1,2-shift of a proton at C3 to C4 led to the formation of ketone **191**. This was observed when BF_3_·Et_2_O, InCl_3_, TfOH, or ZnBr_2_ were used as catalysts. Elimination of a proton from C15 led to formation of alcohol **192** with a second exocyclic double bond in reaction mediated by ZnBr_2_, Bi(OTf)_3_, *p-*TSA or TFA. A 1,2-rearrangement or migration of C2 results in the formation of a cyclopentane derivative **193** in the presence of InCl_3_ or Bi(OTf)_3_.

**Scheme 34:**
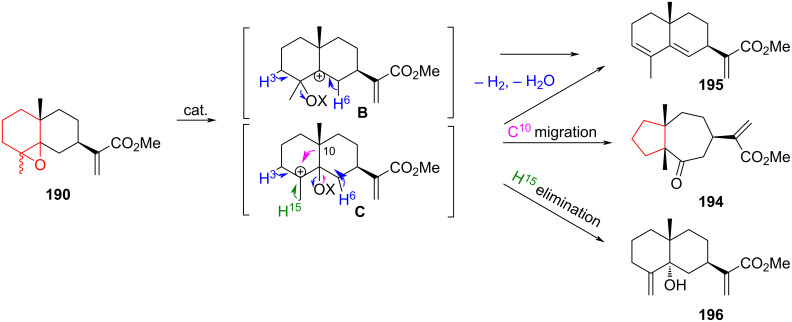
Methods of disclosure of epoxide **190**.

The action of the same Lewis acids on the angular epoxide **190**, a regioisomer of epoxide **189**, led to other products (Scheme 34). According to the authors [87], depending on the nature of the Lewis acid, the reaction proceeded through the formation of intermediate carbocations **B** and **C**. The cyclopentane derivative **194**, annelated with cycloheptanone, was obtained from carbocation **C**. As a result of the reaction, the C10 atom underwent migration, which led to the expansion of the cyclopentane ring. Compound **195** was the product of the double dehydration of carbocations **B** and **C**. The regioisomer of alcohol **192**, a tertiary alcohol **196**, was formed as a result of the elimination of a proton from the C15 atom. It is noteworthy that the reaction of 1,2-oxirane **190** with InCl_3_, ZnBr_2_, TfOH, or Bi(OTf)_3_ resulted in the formation of a mixture of ketone **194** and cyclic diene **195**. Regio- and stereoselectivity was observed in the case of the epoxide **189**. The cyclopentane derivative **194** was obtained stereoselectively and in high yield during the reaction with BF_3_·Et_2_O.

**Scheme 35:**
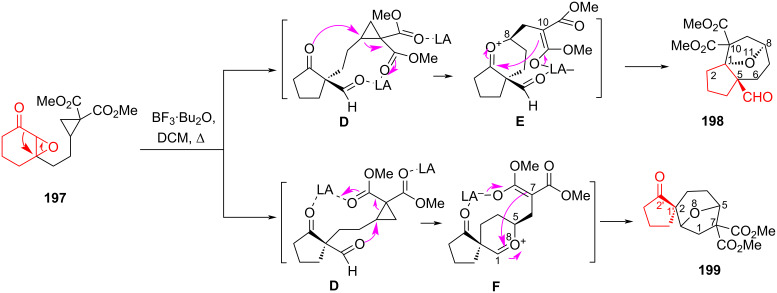
Rearrangement of α,β-epoxy ketone **197**.

An alternative example of the tandem epoxide rearrangement of α,β*-*epoxy ketone **197** using Lewis acids (Sc(OTf)_3_, TMSOTf, SnCl_4_, Yb(OTf)_3_, BF_3_·Et_2_O, BF_3_·CH_3_CO_2_H, BF_3_·THF, BF_3_·Bu_2_O) as a catalyst and different solvents (DCE, DCM, THF, CHCl_3_) has been described in [88]. By varying the temperature, solvent, and catalyst, optimal conditions were found for time, yield, and ratio of reaction products (Scheme 35). The authors [88] suggested that the 1,2-oxirane was opened by the Lewis acid at the first stage. This is accompanied by the contraction of the six-membered ring and the formation of an intermediate **D**. The spatially close reactive centers in molecule **D** promotes tandem transformations with the Lewis acid, resulting in oxygen-containing bicyclic compounds **198** and **199** through the formation of oxonium intermediates **E** and **F**.

**Scheme 36:**
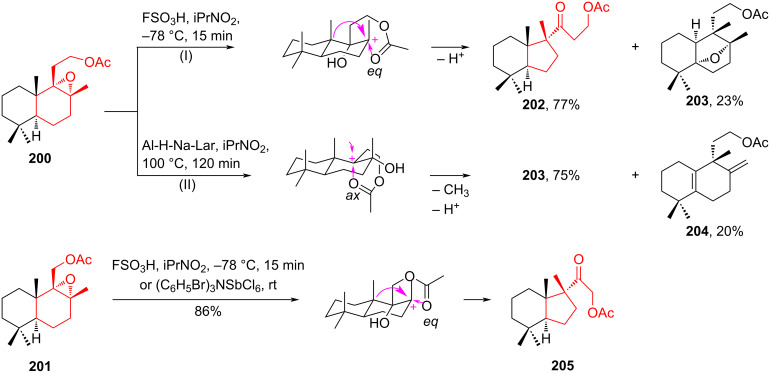
Acid-induced rearrangement in the synthesis of perhydrindane ketones **202** and **205**.

In the work of Kulciţki et al. [89], a very attractive acid-induced rearrangement of homodrimanic epoxide **200** was described. This reaction proceeded through two alternative directions with the formation of ring-contraction products – perhydrindane **202** (pathway I) and oxide **203**, which has a halimanic bicyclic system (pathway II). The use of fluorosulfonic acid as a promoter at low temperature contributed to ring contraction to form the perhydrindanic structure. In contrast, the use of Al-H-Na-Lar pillared clay [90] as a heterogeneous catalyst and heating in 2-nitropropane at 100 °C induced angular methyl migration with formation of the halimanic bicyclic system **203** [90]. The acid-induced rearrangement of the drimanic epoxide **201** proceeded exclusively to the ring-contracted perhydrindanic ketone **205** in high to quantitative yields (Scheme 36). The authors suggest that the selectivity of the acid-induced rearrangement of epoxides **200** and **201**, differing only by a single CH_2_ group, is influenced by the involvement of the lateral chain in the stabilization of cationic reaction intermediates [91–92].

**Scheme 37:**
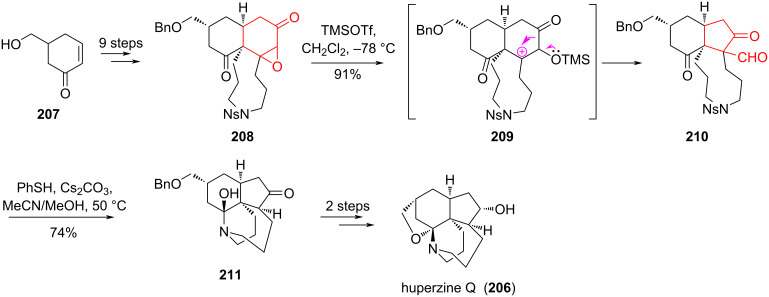
Rearrangement of epoxyketone **208** in the synthesis of huperzine Q (**206**).

Yokoshima and co-workers [93–94] developed an elegant approach to the total synthesis of the alkaloid huperzine Q (**206**) in racemic form (Scheme 37). In the process of synthesis, a *cis*-hydrindane core was prepared from the known hydroxyketone **207** using the Diels–Alder reaction and the ring contraction reaction of epoxyketone **208**. After treatment with TMSOTf in dichloromethane at −78 °C, epoxyketone **208** was subjected to selective cleavage of the epoxide, followed by а 1,2-shift of the carbonyl group and the formation of the ring-contracted ketoaldehyde **210** with a yield of 91%. Under the conditions of removal of the nosyl group, cleavage of the formyl group occurred concomitantly with the addition of methanol to afford hemiaminal **211** with a 74% yield. Subsequent two-step synthetic transformations of **211** yielded huperzine Q (**206**) in 50% yield.

**Scheme 38:**
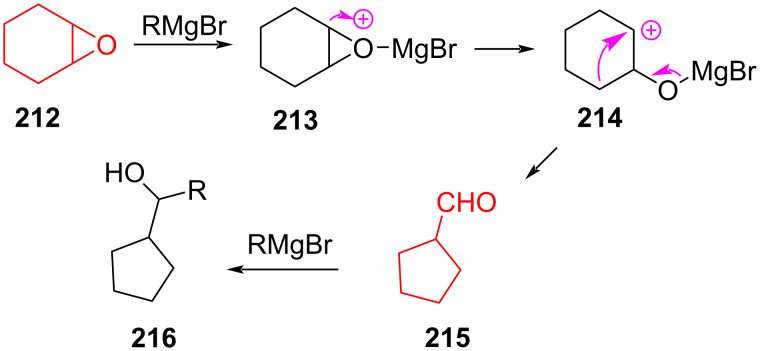
Rearrangement of epoxide **212** under the action of Grignard reagent.

Banerjee et al. [95] demonstrated an alternative method for the synthesis of cyclopentane derivatives. The method included the cleavage of epoxides using Grignard reagents followed by ring contraction. Treatment of epoxide **212** with Grignard reagent through intermediates **213** and **214** produced aldehyde **215**, and further reaction of **215** with the Grignard reagent gave alcohol **216** (Scheme 38).

**Scheme 39:**
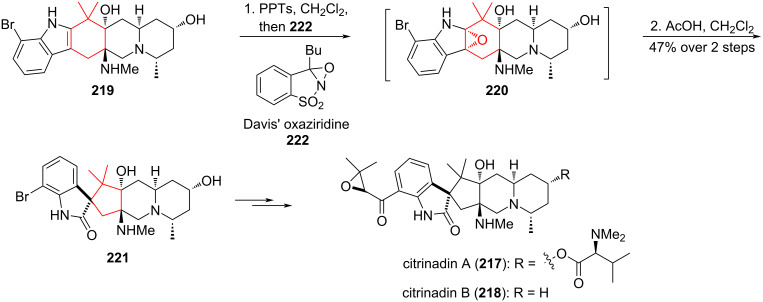
Semipinacol rearrangement of epoxide **220** in the synthesis of (−)-citrinadin A (**217**) and (+)-citrinadin B (**218**).

A promising method for the contraction of cyclohexane rings is the semipinacol rearrangement. For the enantioselective total synthesis of (−)-citrinadin A (**217**) and (+)-citrinadin B (**218**), the authors [96] used the semipinacol rearrangement as a key step in converting indole **219** to spirooxindole **221**. The spirooxindole alkaloids, (−)-citrinadin A and (+)-citrinidin B, exhibit notable activity against murine leukemia L1210 (**217**, IC_50_ 6.2 μg/mL; **229**, 10 μg/mL) and human epidermoid KB cells (**218**, IC_50_ 10 μg/mL). Sequential interaction of indole **219** with pyridinium *p*-toluenesulfonate (PPTS) and excess of Davis’ oxaziridine **222**, an effective oxidizing agent for synthesis of spirooxindole alkaloids, led to epoxide **220**. Labile oxirane **220**, without isolation, was subjected to a semipinacol rearrangement with acetic acid. The yield of spirooxindole **221** as a single stereoisomer was 49% (Scheme 39).

**Scheme 40:**
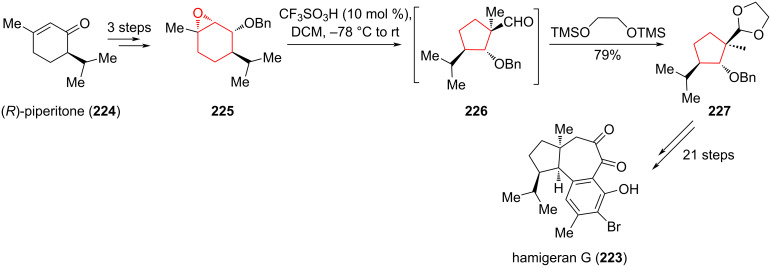
Semipinacol rearrangement of epoxide **225** in the synthesis of hamigeran G (**223**).

In 2016 Gao and co-workers [97], reported the first total synthesis of the halogenated tetraterpenoid hamigeran G (**223**), which inhibited growth of the P388 tumor cell line and the HL-60 promyelocytic leukemia cell line (IC_50_ 8 μM). The key step in this approach was the acid-promoted semipinacol rearrangement of epoxide **225** under the action of trifluoromethanesulfonic acid to produce aldehyde **226** with a quaternary carbon atom (C9) as a single diastereomer. The addition of 1,2-bis(trimethylsiloxy)ethane to the reaction mixture protected the aldehyde group as a dioxolane, providing cyclopentane **227** with a yield of 79% in two steps (Scheme 40).

**Scheme 41:**
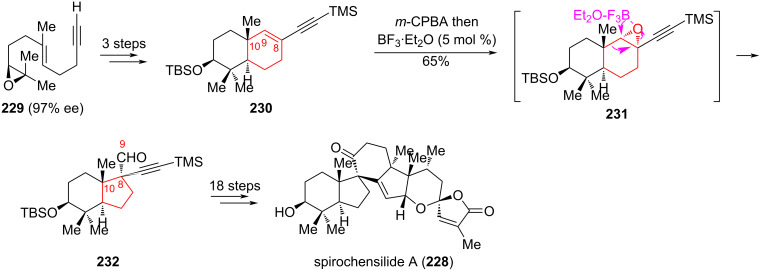
Semipinacol rearrangement of epoxide **23**1 in the synthesis of (−)-spirochensilide A (**228**).

In the alternative stereoselective synthesis of (−)-spirochensilide A (**228**), proposed by Liang et al. [98], one of the key steps was the semipinacol rearrangement of alkyne **230** into aldehyde **232**, which contains two vicinal quaternary chiral centers at C8 and C10 (Scheme 41). (−)-Spirochensilide A belongs to an emerging and biologically important class of natural products with a unique spirocyclic core [99–102]. It is also a promising compound for the study of inflammatory diseases and other bioactive properties. Epoxidation of **230** with *m*-chloroperoxybenzoic acid *(m-*CPBA) gave the epoxide **231**. Subsequent addition of excess BF_3_·OEt_2_ led to a semipinacol rearrangement, affording **232** in 65% yield as a single diastereomer. The aldehyde **232** was then converted by a series of synthetic transformations to (−)-spirochensilide A (**228**) with a total yield of 2.2% in 22 steps starting from acetylenic epoxide **229**.

#### 4.1 Wagner–Meerwein rearrangement

The isomerization of terpenes via cleavage, addition or nucleophilic substitution reactions accompanied by a redistribution of C–C bonds in the ring, is called the Wagner–Meerwein rearrangement. Although this rearrangement has been known since the 19th century, only a limited number of articles have been published about it in the last decade.

**Scheme 42:**
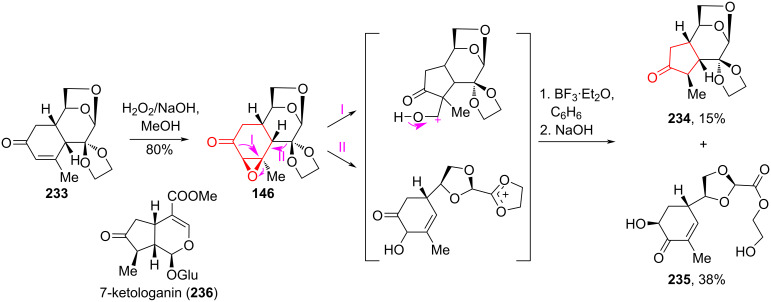
Wagner–Meerwein rearrangement in the synthesis of compound **234** with iridoid topology.

The Wagner–Meerwein and Favorskii contraction of a cyclohexene ring was studied by Faizullina et al. [76] using the epoxy derivative of enone **233**. The required α-ketooxirane **146** was obtained from enone **233** by oxidation with a mixture of 30% H_2_O_2_ and NaOH in methanol with a yield of 80% (Scheme 42). After treatment of α-ketooxirane **146** with BF_3_·Et_2_O, two compounds, cyclopentanone **234** and the Grob fragmentation product, enone **235**, were isolated from the reaction mixture. Cyclopentanone **234** can be used in the synthesis of 7-ketologanin (**236**) after optimization of the transformation. The product of the Wagner–Meerwein rearrangement, cyclopentanone **234**, was formed according to the classical mechanism, and the process was completed by decarbonylation. Enone **235** was obtained by concurrent Grob fragmentation through an intermediate 1,3-dioxolan-2-ylium ion.

**Scheme 43:**
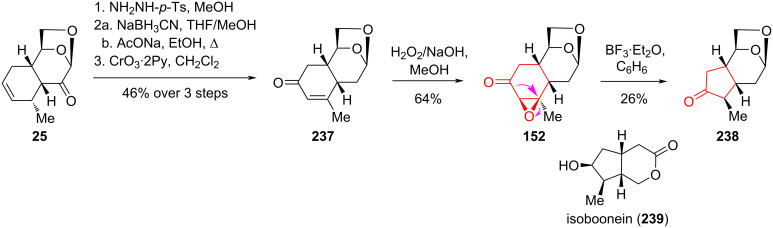
Wagner–Meerwein rearrangement in the synthesis of compound **238** with iridoid topology.

In order to eliminate the influence of the dioxolane fragment on the transformation process, the same authors [76] deoxygenated ketone **25** by transforming it to the corresponding tosylhydrazone, followed by deamination to give derivative **237** [103]. This derivative was then converted to epoxide **152** using H_2_O_2_–NaOH in methanol (Scheme 43). Subsequent treatment of compound **152** with BF_3_·Et_2_O in benzene afforded the annelated methylcyclopentanone **238** with a yield of 26%. This compound can be used in the synthesis of isoboonein (**239**) [104].

**Scheme 44:**
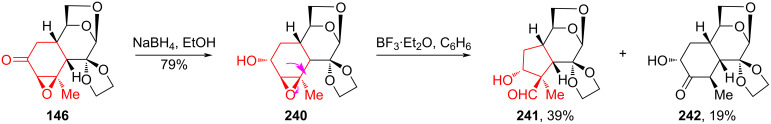
Wagner–Meerwein rearrangement in the synthesis of compound **241** with iridoid topology.

In compound **146**, the effect of the keto group is more significant than that of the dioxolane fragment, as is known for this type of compounds. Therefore, changes to this functionality or its removal can affect the course of reactions. To clarify this issue, α-hydroxyoxirane **240** was stereospecifically obtained by borohydride reduction of α-ketooxirane **146**. Subsequent treatment with BF_3_·Et_2_O resulted in the formation of aldehyde **241** with a yield of 39% and hydroxyketone **242** with a 19% yield (Scheme 44). An attempt to perform similar procedures on α-ketooxirane **152** lacking the the dioxolane fragment, led, as expected, to the formation of a difficult-to-separate mixture of products. It is interesting to note that in the studied series of oxiranes, derivatives of the Diels–Alder adduct of LG and piperylene, the structure of α-hydroxy derivative **240** was found to be most optimal for the Wagner–Meerwein rearrangement under the action of BF_3_·Et_2_O to an annelated cyclopentane derivative.

**Scheme 45:**
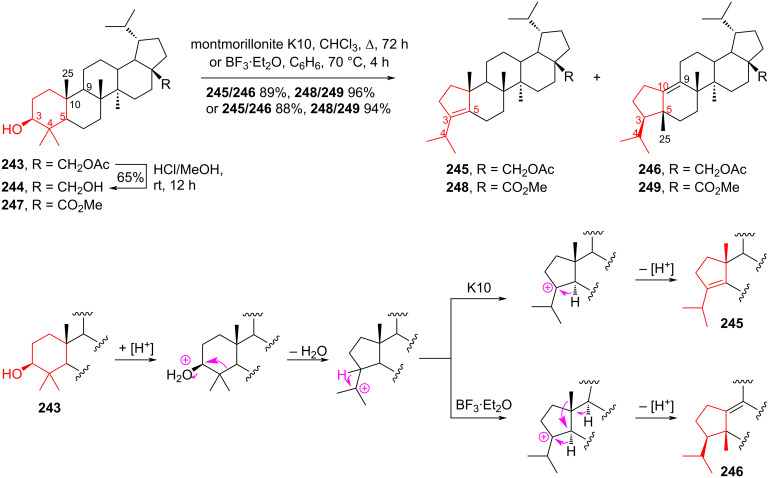
Wagner–Meerwein rearrangement in the synthesis of lupane derivatives **245**, **246, 248**, and **249**.

In 2020, Pakulski and co-workers [105] applied a convenient ring-contraction strategy using the Wagner–Meerwein rearrangement to lupane derivatives containing the betulin framework. This reaction was induced by a number of catalysts, including hydrogen chloride, montmorillonite K10, and boron trifluoride etherate. As a result, promising derivatives for the synthesis of ceanothic acid analogues were obtained [106–109]. When treating betulin monoacetate **243** with hydrogen chloride in methanol, the deacetylation of the product to dihydrobetulin **244** was observed with a yield of 65%. Ring contraction was observed when compound **243** was treated with montmorillonite K10 in boiling chloroform, giving an unseparated mixture of abeolupanes **245** and **246**, in a ratio of 1.5–3:1, with a total yield of 89%. The interaction of compound **243** with BF_3_·Et_2_O in benzene at 70 °C gave a mixture of olefins **245** and **246** in a 1:2 ratio, with an overall yield of 88%. Similar transformations were carried out with methyl dihydrobetulinate **247**. In the presence of montmorillonite K10, a ring contraction was observed to form an unseparated mixture of olefins **248** and **249** at a ratio of 2–2.5:1, with a total yield of 96%. A highly selective rearrangement was observed when BF_3_·Et_2_O was present to form olefins **249** and **248** in a ratio of >4:1, with a yield of 94% (Scheme 45).

**Scheme 46:**
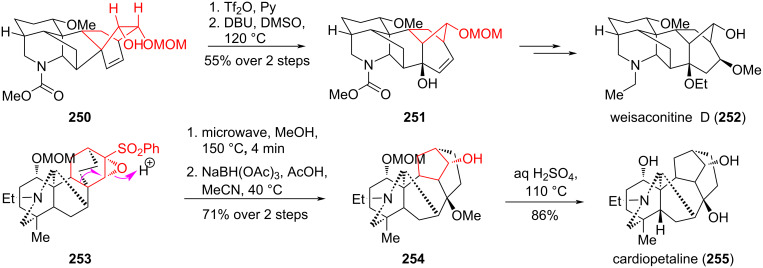
Wagner–Meerwein rearrangement in the synthesis of weisaconitine D (**252**) and cardiopetaline (**255**).

The Wagner–Meerwein rearrangement is widely used in the synthesis of diterpenoid alkaloids of the aconitine-type (Scheme 46). Sarpong et al. [110] used the triflate derived from alcohol **250** as a substrate for the Wagner–Meerwein rearrangement, leading to the synthesis of weisaconitine D (**252**) and lilestrandinin. In 2016, Nishiyama et al. synthesized cardiopetaline (**255**) through the rearrangement of sulfonyloxirane **253** [111].

**Scheme 47:**
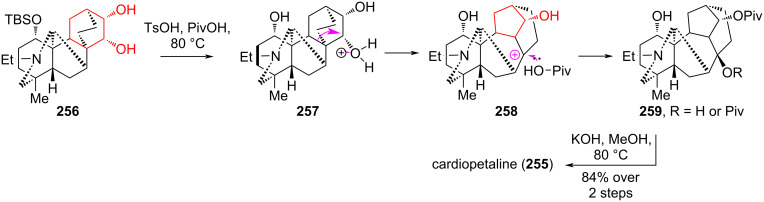
Wagner–Meerwein rearrangement in the synthesis of cardiopetaline (**255**).

In 2017, Nishiyama et al. presented an elegant synthesis of cardiopetaline (**255**) by converting the denudatine skeleton to the aconitine skeleton via the Wagner–Meerwein rearrangement of diol **256** [112]. The rearrangement was initiated by heating **256** with *p-*toluenesulfonic acid (TsOH) in pivalic acid (PivOH) to form a mixture of pivalates **259**. The hydrolysis of this mixture allowed cardiopetaline (**255**) to be obtained with a yield of 84% (Scheme 47). This strategy does not require preactivation of the pivotal hydroxy group, the differentiation of several hydroxy groups in the polyoxygenated substrate molecule, and can be applied to the synthesis of highly oxygenated diterpenoid alkaloids of the aconitine-type.

## Conclusion

An analysis of literature data over the last 12 years has shown that ring-contraction reactions are widely used in the synthesis of various natural compounds and their structural analogues, which are useful for creating compound libraries. Indeed, ring interconversions play an important role in organic synthesis, allowing the creation of highly functional small carbocycles with high numbers of stereocenters, thereby being a powerful method for the total synthesis of biologically active compounds. The advantages of transformations and ring rearrangements are the reduction of synthetic steps, construction of complex structures of the reaction products, and use of chiral substrates, which solve the problem of controlling optical activity. We hope this review will serve as a stimulus for the further application of well-established ring-contraction reactions and inspire colleagues to develop new, practical and convenient strategies.

## Data Availability

Data sharing is not applicable as no new data was generated or analyzed in this study.
